# Supplementary material for: Risk factors for sacrococcygeal pilonidal sinus: a systematic review and meta-analysis supplemented by genetic causal assessment
Source: Front Surg. 2026 Jan 7;12:1718589. doi: 10.3389/fsurg.2025.1718589 (PMC12819706; doi:10.3389/fsurg.2025.1718589)
Supplement: Supplementary file 2 [file Datasheet2.zip › Supplementary Data 2/MR_pipeline_p5e-6/leaveone_plots/ebi-a-GCST006095.finngen_R12_L12_PILONIDALCYST.pdf]

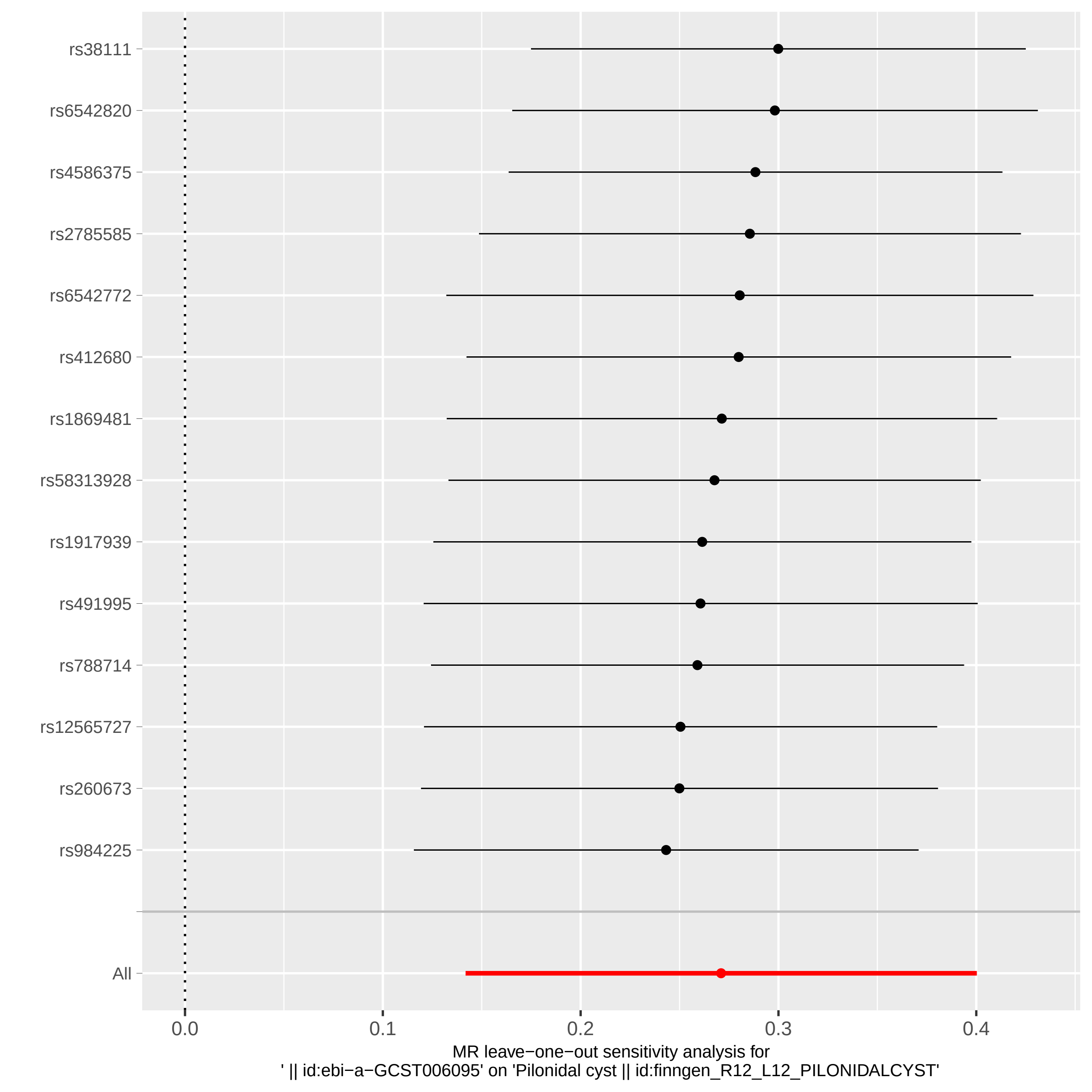
rs38111
rs6542820
rs4586375
rs2785585
rs6542772
rs412680
rs1869481
rs58313928
rs1917939
rs491995
rs788714
rs12565727
rs260673
rs984225
All
0.0
0.1
0.2
0.3
0.4
MR leave−one−out sensitivity analysis for
' || id:ebi−a−GCST006095' on 'Pilonidal cyst || id:finngen_R12_L12_PILONIDALCYST'
